# A Methodological Advance of Tobacco Rattle Virus-Induced Gene Silencing for Functional Genomics in Plants

**DOI:** 10.3389/fpls.2021.671091

**Published:** 2021-06-04

**Authors:** Gongyao Shi, Mengyuan Hao, Baoming Tian, Gangqiang Cao, Fang Wei, Zhengqing Xie

**Affiliations:** ^1^Zhengzhou Research Base, State Key Laboratory of Cotton Biology, Zhengzhou University, Zhengzhou, China; ^2^School of Life Sciences, Zhengzhou University, Zhengzhou, China; ^3^Henan International Joint Laboratory of Crop Gene Resources and Improvements, School of Agricultural Sciences, Zhengzhou University, Zhengzhou, China

**Keywords:** TRV-VIGS, vector construction, agroinfiltration, secondary inoculation, methodology modification

## Abstract

As a promising high-throughput reverse genetic tool in plants, virus-induced gene silencing (VIGS) has already begun to fulfill some of this promise in diverse aspects. However, review of the technological advancements about widely used VIGS system, tobacco rattle virus (TRV)-mediated gene silencing, needs timely updates. Hence, this article mainly reviews viral vector construction, inoculation method advances, important influential factors, and summarizes the recent applications in diverse plant species, thus providing a better understanding and advice for functional gene analysis related to crop improvements.

## Introduction

Virus-induced gene silencing (VIGS) is a high-throughput reverse genetics technique that exploits an RNA-mediated antiviral defense mechanism [post-transcriptional gene silencing (PTGS)] for functional gene analysis (Ratcliff et al., 1997; Sunilkumar et al., 2006). VIGS was originally used to describe the recovery of viral symptoms on plants after virus infection (Kammen, 1997). Subsequently, researchers demonstrated that this is a manifestation of the plant’s natural defense mechanism induced by virus infection, and some endogenous genes that are homologous to viral genomes could also be silenced at the same time (Ratcliff et al., 1999). Thus, scientists modified the viral genome [complementary DNA (cDNA)] into a recombinant virus vector containing sequences that were homologous to host genes, which could trigger homologous endogenous gene silencing in plants.

**FIGURE 1:**
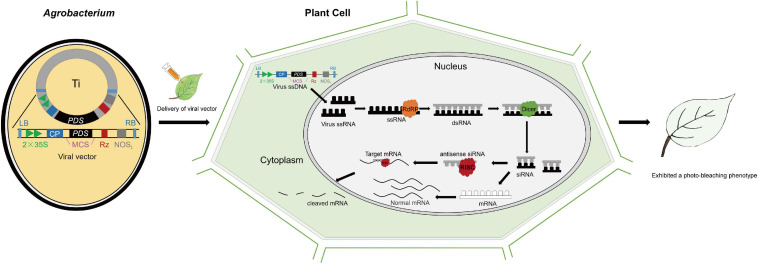
Virus-induced gene silencing (VIGS) mechanism. *Phytoene desaturase* (*PDS*) serves as an example. Upon infection, the T-DNA carrying the viral genome is transformed into the plant by *Agrobacterium* and then transcribed by the host’s RNA polymerase. RNA-dependent RNA Polymerase (RdRP) (yellow) produces double-stranded RNA (dsRNA) from the single-stranded RNA (ssRNA) viral transcript. The dsRNA is then recognized by DICER-like enzyme, Dicer (green) and cleaved into short interfering RNAs (siRNAs). Antisense siRNAs are recognized by RNA-induced silencing complex, RISC (red) and melted into ssRNAs, which then serve as templates for target gene degradation. The single-stranded siRNAs are amplified and spread as mobile silencing signals throughout the plant, thus resulting in target gene silencing in plant organs distant from the site of infection, symbolized by the photo-bleaching phenotype of the entire leaf. LB: left border; RB: right border; 2 × 35S: duplicated cauliflower mosaic virus 35S promoter; CP: coat protein; MCS: multiple cloning site; *PDS*: *PDS* cDNA fragment; Rz: self-cleaving ribozyme; NOS_*t*_: nopaline synthase terminator.

*Phytoene desaturase* (*PDS*) and *Agrobacterium*-mediated tobacco rattle virus (TRV)-VIGS by leaf injection can be used as an example (Figure 1). Recombinant vectors containing 300–500 bp (base pair) cDNA fragments and devoid of homopolymeric regions of the *PDS* gene were introduced into plant cells during agroinfiltration with *Agrobacterium* cultures. After agroinfiltration, the T-DNA, including the viral genome, was transcribed into sing-strand RNA (ssRNA) in the host plant cells. Then, large amounts of double-stranded RNAs (dsRNAs) generated by RNA-dependent RNA polymerase (RdRp) encoded by the viral genome were detected by the host plant genome as aberrant sequences, and thus cleaved into short interfering RNA (siRNA) duplexes of 21–24 nucleotides by the action of Dicer-like proteins (Ding and Voinnet, 2007). SiRNAs were incorporated as single-stranded RNAs into RISC (RNA-induced silencing complex), which specifically screens and destroy the mRNAs (*PDS* transcripts) complementary to the siRNAs, resulting in degradation of mRNAs and a photo-bleaching plant phenotype (Waterhouse et al., 2001; Baulcombe, 2002; Zhang et al., 2014; Figure 1).

Compared with other traditional genetic tools, VIGS can rapidly (in 3–4 weeks) silence endogenous genes and display an easily observed silenced phenotype in contemporary plants, with no need for stable transformants. Partial sequence information is sufficient to silence the target gene, and there is a simple operation procedure. Therefore, VIGS technology has been widely used as a high-throughput genetic tool for genetic screening and functional genomics in many species (Lu et al., 2003; Burch-Smith et al., 2004), such as tomato (Liu et al., 2002a), tobacco (Senthil-Kumar and Mysore, 2011b), soybean (Liu et al., 2015), wheat, and corn (Zhang et al., 2017). Methodological advances of VIGS are very important in the post-genomic era. In this review, we firstly summarize the development history of the vector construction and inoculation methodology, and then focus on the significant efficiency influential factors in VIGS application, as well as the adoptable range of plant species of TRV-VIGS is updated here. Therefore, this review would provide a better reference for the methodological study of TRV-VIGS and functional analysis related to genetic improvements in crop breeding.

## TRV-VIGS Vector Construction

**FIGURE 2:**
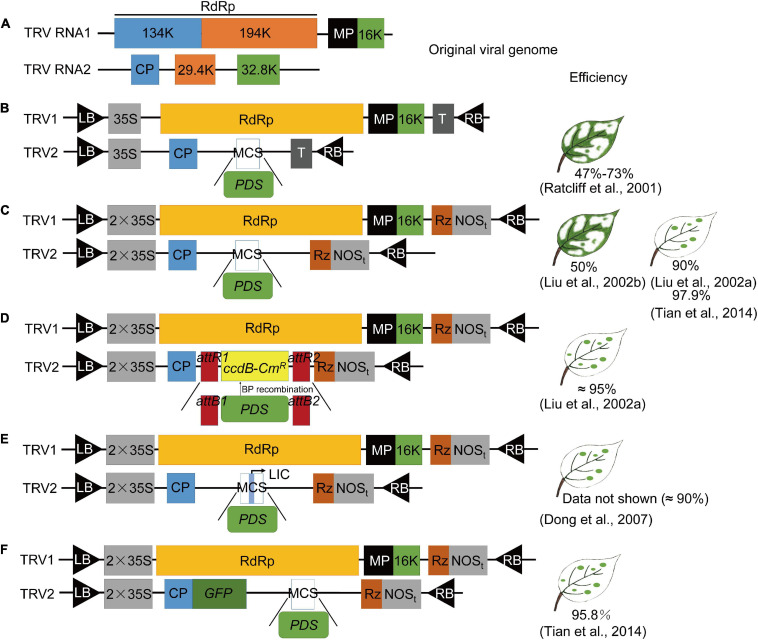
The development process of TRV vector construction. *Phytoene desaturase*, *PDS* was used as an example. **(A)** Genome organization of tobacco rattle virus (TRV). The TRV1 open reading frames (ORFs) correspond to 134 and 194 kDa replicases, a movement protein (MP), and a 16 kDa cysteine-rich protein (16K). The TRV2 ORFs correspond to the coat protein (CP) and 29.4 and 32.8 kDa proteins (29.4K and 32.8K). **(B)** TRV-based VIGS vector (Ratcliff et al., 2001). Ratcliff et al. constructed separate cDNA clones of TRV (strain PPK20) RNA1 and RNA2 under the control of cauliflower mosaic virus (CaMV) 35S promoters on the transferred (T) DNA of plant binary transformation vectors. They replaced the non-essential 29.4K and 32.8K genes with a multiple cloning site (MCS), leaving only the 5′ and 3′ untranslated regions and the viral coat protein. The cDNA clones were positioned between the left and right border (LB and RB) of the T-DNA and between CaMV 35S promoters (35S) and transcriptional terminators (T). The TRV open reading frames corresponded to the RdRp, MP, 16K, CP, and 29.4K and 32.8K proteins. **(C)** TRV-based VIGS vector (Liu et al., 2002a,b; Tian et al., 2014). TRV1: TRV cDNA clones were placed between the duplicated CaMV 35S promoter (2 × 35S) and the nopaline synthase terminator (NOS_*t*_) in a T-DNA vector. Rz, self-cleaving ribozyme. TRV2: TRV cDNA clones were placed between the duplicated CaMV 35S promoter (2 × 35S) and the nopaline synthase terminator (NOS_*t*_) in a T-DNA vector, and *PDS* was added to the MCS between CP and Rz. **(D)** Modified pTRV2 vector based on GATEWAY cloning technology containing attR1 and attR2 recombination sites (Liu et al., 2002a). The PCR products flanked by attB1 and attB2 sequences directionally recombined *in vitro* at the attR1 and attR2 sites contained in the plasmid when incubated with the BP CLONASE enzyme. Then, the *PDS* gene was cloned into the pTRV2-attR1-attR2 vector. **(E)** TRV-Ligation-independent cloning (LIC) vector (Dong et al., 2007). The TRV-LIC vector was created by inserting a cassette, containing adapters and two *Pst*I sites, in two digestion and ligation reactions. Then, the CDS insely digested enzyme for digestion and ligation. **(F)** TRV-GFP vector (Tian et al., 2014). GFP CDS was fused with CP in the TRV2 vector to generate an easily traceable TRV vector in different parts of plants.

Kumagai et al. (1995) constructed the first VIGS vector based on tobacco mosaic virus (TMV); they successfully knocked down *NbPDS* gene expression and obtained *NbPDS* silenced plants with an albino phenotype by inoculating *in vitro* RNA transcripts into *Nicotiana benthamiana*. Subsequently, many viral vectors were successfully modified and used for VIGS studies (Supplementary Table 1). RNA viruses were the earliest and most widely used viral carrier for the establishment of the VIGS system because of their small molecular weight and high infection efficiency (Bruun-Rasmussen et al., 2007). DNA viruses account for a small number of plant viruses with a large genome structure and limited movement in plants. Satellite viruses do not cause any diseases in plants themselves; they are generally not associated with any illness or interference with the true gene silenced phenotype. However, they are only suitable for use in a small number of host plants (Zhou et al., 2012; Supplementary Table 1). Most viruses used for VIGS cannot infect the plant growing points or meristems (Ratcliff et al., 2001; Harper et al., 2002; Supplementary Table 1), but the TRV-VIGS system successfully overcomes the host limitations of meristem transmission (Ratcliff et al., 2001; Liu et al., 2002b) by (1) effectively spreading to all plant tissues, including the meristems; (2) a wide host range [50 or more families (Solanaceae (Purkayastha and Dasgupta, 2009), Cruciferae (Zhang et al., 2004), and Gramineae (Scofield et al., 2005)), among others, in dicots and monocots]; and (3) developing mild viral symptoms after infection. Therefore, TRV vectors have been widely used for VIGS studies, and the viral vector modification of TRV that is important for silencing efficiency of the VIGS system is reviewed (Figure 2).

Tobacco rattle virus is a positive-sense RNA virus composed of the RNA1 and RNA2 genome. RNA1 encodes two replicases and one cysteine-enriched protein, which is sufficient for replication and movement within the plant even without RNA2. One coat protein (CP) and two non-structural proteins encoded by RNA2 allow virion formation and nematode-mediated transmission across plants (Ratcliff et al., 1999; Liu et al., 2002b; Figure 2A). Ratcliff et al. (1999) first used *in vitro* transcripts of the cDNA clones of TRV (strain PPK20) RNA1 and modified RNA2 [TRV-*Green* fluorescent protein (GFP) RNA2]. GFP was transcribed from the coat protein promoter (P) of the pea early browning virus and replaced the 29.4K (29.4 kDa) and 32.8K (32.8 kDa) open reading frames (ORF) to express *GFP* successfully in tobacco. Then, they constructed the original TRV-VIGS vector; the complete cDNA sequences of RNA1 and RNA2 were positioned between the left and right border (LB and RB) of the T-DNA (pBINTRA6) and between cauliflower mosaic virus (CaMV) 35S promoters (35S) and transcriptional terminators (T), and a multiple cloning site (MCS) was introduced during the cloning of RNA2 (TRV00, Ratcliff et al., 2001; Figure 2B). They inserted partial cDNA sequences of the *NbPDS* gene into MCS of TRV00, and successfully silenced *NbPDS* with mild viral symptoms and a silencing efficiency of 47–73%. In the following year, Liu et al. (2002a, b) developed the most commonly used TRV vector (TRV2-MCS, pYL156; Figure 2C) by using the duplicated CaMV 35S promoter (2 × 35S), instead of a single 35S, and adding a self-cleaving ribozyme (Rz, to increase virus infectivity) before the nopaline synthase terminator (NOSt) on the transferred *Agrobacterium tumefaciens* T-DNA of plant binary transformation vectors, which had a high silencing efficiency (90–97.9%). They successfully studied the function of tobacco RAR1 and other genes against TMV resistance (Liu et al., 2002a,b; Tian et al., 2014).

As TRV2-MCS is a labor-intensive and time-consuming cloning-dependent method, the same team modified the pTRV2 clone into a GATAWAY recombination system (TRV2-GATEWAY, pYL279), which allowed fast and easy cloning that was free of the restriction enzyme and ligation and could be used for large-scale functional genomics analysis (Liu et al., 2002a; Figure 2D). The construction of the recombinant TRV vector requires two or more cloning steps (Ratcliff et al., 2001). Thus far, compared with other VIGS systems, the gene silencing efficiency of TRV-MCS and TRV-GATEWAY in *N. benthamiana* has been widely adopted, and with these vectors, scientists successfully silenced the *PDS* gene in tomato and other species with a silencing efficiency around 90% (Liu et al., 2002a; Tian et al., 2014). These two improved TRV-VIGS vectors have been the most commonly used vectors for gene-functional studies (Chen et al., 2004; Senthil-Kumar and Mysore, 2011b; Liu et al., 2015; Zhang et al., 2017).

A new TRV2-Ligation-independent cloning (LIC) vector (pYY13) in which the inserts can be cloned independently of the connection (LIC), instead of using the expensive GATEWAY-based recombination system, has been developed (Dong et al., 2007; Figure 2E). The TRV2 vector, pYL170, which is identical to pYL156 (except for a plant selection marker), was used to generate the new TRV2-LIC vector by inserting a ccdB cassette containing adapters and two *Pst*I sites using two digestion and ligation reactions (Burch-Smith et al., 2006). The insert requires the addition of adapter sequences, and together with pYY13, after treated with T4 DNA polymerase, both can be transformed into *Escherichia coli* for construction of the recombinant vector (Dong et al., 2007; Figure 2E). Compared with the commonly used TRV-MCS, the TRV-LIC vector is more efficient (about 90%). Therefore, the TRV-LIC vector allows high-throughput cloning of silenced fragments without the use of expensive recombinases (Dong et al., 2007).

With the deepening of TRV-VIGS application, researchers attempted to determine whether virus-infected and -affected sites could be monitored in more species. Tian et al. (2014) added the full cDNA sequence of *GFP* to the 3′ terminus of coat protein in the original TRV2 vector (pYL156) to form a fusion protein of CP and GFP and successfully tested the modified vector (Figure 2F) in many plants, including *N. benthamiana*, *Arabidopsis thaliana*, *Rosa rugosa Thunb*, and *Fragaria ananassa*. The proportion of TRV-GFP-PDS positive silenced plants (95.8%) was very close to that of TRV-PDS-VIGS positive plants (TRV2-MCS, 97.9%) in *N. benthamiana*, and the same phenotype could be replicated in other plants, indicating that the insertion of GFP did not change the gene silencing ability of TRV vectors (Tian et al., 2014). Transmission of the modified TRV-GFP virus can be easily detected using a fluorescence microscope and a handheld UV lamp. This improved TRV vector is a simple but visualizable and efficient genetic tool for functional genomics, especially in non-Solanaceae plants (Tian et al., 2014).

Apart from these vector construction modifications, there were also other studies about TRV-VIGS, like silencing multiple genes of Arabidopsis by inserting tandem gene sequences into TRV-MCS vector (Burch-Smith et al., 2006), silencing tandem constructs of the TRV-GATEWAY vector containing CHS (chalcone synthase) as a reporter gene to exam the function of floral-associated genes in petunia (Chen et al., 2004), or successfully elucidating the role of 24 highly homologous *N. benthamiana* ubiquitin E2 gene family members in plant immunity through extension PCR of different conserved gene fragments together with the TRV-GATEWAY system (Zhou and Zeng, 2017). TRV1 has also been modified for inserting plant gene fragments and invoking gene silencing without TRV2 (Deng et al., 2013). Taken together, these studies have contributed to the versatility of TRV-VIGS in plants, and more efficient TRV-based VIGS systems are expected to be developed in the future.

## Methodology Progress of TRV-VIGS

**FIGURE 3:**
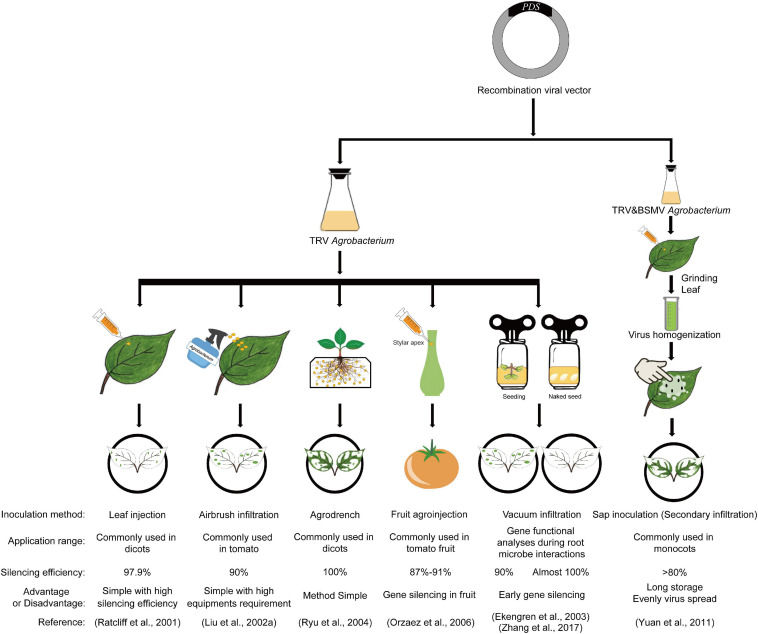
Schematic diagram of different VIGS inoculation methods. The *phytoene desaturase*, *PDS* gene was used as an example. Inoculation method, application range, silencing efficiency, advantages and disadvantages, and corresponding references are all included. TRV: Tobacco rattle virus; BSMV: Barley stripe mosaic virus.

The delivery of the constructed recombinant viral vector carrying partial cDNA sequences of target genes into plants for gene silencing is always very important for the efficacy of VIGS; therefore, scientists have developed many ways to introduce the viral vectors into plants. Initially, Ruiz et al. (1998) delivered the infectious potato virus X (PVX) RNA transcripts obtained by *in vitro* transcription into plants by mechanically rubbing them onto the leaves of 4- to 5-week-old wild-type *N. benthamiana* in the presence of a small amount of carborundum and successfully obtained the *NbPDS* gene silenced phenotype. However, the *in vitro* transcription of the viral cDNA was difficult and tedious to operate, and the stability of silencing efficiency was sometimes low in VIGS experiments. Later, researchers attempted to transform the recombinant viral vector into *A. tumefaciens*, which is better for viral cDNA transcription and is also sufficient for the exploration of the interaction between plants and viruses, and they successfully silenced target genes by syringe infiltration (Ratcliff et al., 2001). To make full use of TRV-VIGS in more species, many agroinfiltration methods were developed (Figure 3), such as leaf injection (syringe infiltration), *Agrobacterium* spray (airbrush infiltration), agrodrench, fruit agroinjection, vacuum infiltration, and even secondary inoculation by *N. benthamiana* leaf sap after inoculation with *Agrobacterium* cultures (Ratcliff et al., 2001; Liu et al., 2002a; Ekengren et al., 2003; Ryu et al., 2004; Hileman et al., 2005; Chung et al., 2006; Orzaez et al., 2006; Yuan et al., 2011; Figure 3). In this part, the inoculation method improvement of TRV-VIGS is reviewed to provide advice and new dimensions for future methodology studies of VIGS.

### Leaf Injection

Leaf injection (syringe infiltration) is the most common agroinfiltration method used for leaf or floral development-related gene studies in dicots with high silencing efficiency (Figure 3). Ratcliff et al. (2001) silenced the *NbPDS* gene in tobacco by infecting *N. benthamiana* leaves with a 2-ml needleless syringe carrying TRV *Agrobacterium* cultures, and then first proposed this method for VIGS. Liu et al. (2002b) successfully silenced the tomato *PDS* gene with a silencing efficiency of 50% by leaf injection (Liu et al., 2002b). Then, this method with little improvement and higher efficiency was widely used in Arabidopsis (Zhang et al., 2004), tomato (Ekengren et al., 2003), pepper (Choi et al., 2007), and cotton (Gao et al., 2011), among other dicotyledonous plants (Liu et al., 2015), for functional analysis of genes involved in plant (Lu et al., 2011) and floral development (Chen et al., 2005). However, it is laborious for large-scale screening, and monocotyledonous plants are difficult to infiltrate with this method; thus, new methods were developed for VIGS.

### Spray Inoculation

Spray inoculation is mainly suitable for the VIGS of tomato genes with a success rate greater than 90% (Liu et al., 2002a; Dinesh-Kumar et al., 2003; Figure 3). In 2002, by transforming the recombinant TRV-VIGS plasmid into *Agrobacterium* and spraying the *Agrobacterium* cultures into tomato leaves with an art spray gun connected to a portable air compressor set at 75 psi, Liu et al. (2002a) successfully obtained the *SlPDS* silenced phenotype in tomato with a silencing efficiency of 90% (Liu et al., 2002a). Lu et al. (2011) successfully studied the function of the *VTE1* (Tocopherol cyclase) gene involved in tomato vitamin E synthesis with this method (Lu et al., 2011). As the leaves of monocot plant are difficult to be injected with needleless syringe, spray inoculation and leaf friction methods are more adoptable for the VIGS studies of monocotyledonous genes (Dinesh-Kumar et al., 2003).

### Agrodrench

Apart from leaf injection and spray inoculation, researchers also developed other methods to study early root development genes (Figure 3). By drenching the crown region (the soil adjacent to the roots) of young *N. benthamiana* seedlings with 3–5 ml of the TRV-containing *Agrobacterium* cultures (OD_600_ = 1.0), Ryu et al. (2004) successfully silenced the *NbPDS* gene in *N. benthamiana* and named the modified VIGS method agrodench (a simple, efficient, and fast inoculation method for *Agrobacterium* in VIGS experiments) (Ryu et al., 2004; Senthil-Kumar and Mysore, 2011b, 2014). This method is mainly used in diverse Solanaceous plants at the young seedlings stage and is more efficient than the leaf infiltration method for defining the roles of genes in roots (Ryu et al., 2004). The success rate is near 100% in *N. benthamiana* and about 60–70% in tomato, *Nicotiana tabacum* (tobacco), *Petunia hybrida*, *Solanum tuberosum* (potato), *Capsicum annuum* (pepper), and *Solanaceae* (eggplant) (Ryu et al., 2004). This indicates that the combination of different methods of agroinoculation at different stages may be a better choice for triggering heritable gene silencing in plants, which is very necessary and important for longer duration VIGS studies related to crop breeding or other fields.

### Fruit Agroinjection

In addition to the previous infection methods carried out on leaves or roots, researchers also developed a method to study fruit genes. A transient methodology (fruit agroinjection) proposed by Orzaez et al. (2006) gently injected *Agrobacterium* cultures containing partial *SlPDS* cDNA into the fruit through the fruit stylar apex with a syringe, resulting in complete fruit infiltration with a silencing efficiency of 87–91% in tomato (*Solanum lycopersicum*) (Figure 3). This technology was not only good for gene silencing but also as a tool for fast transient expression in fruit (Orzaez et al., 2006), thus increasing the number of studies on fruit development and reproduction in different crops.

### Vacuum Infiltration and Seed Imbibition

Vacuum infiltration is a timesaving agroinoculation method that can be carried out even at the plant seed stage, expanding the range of genes that can be studied by VIGS with a success rate of 90–100% (Ekengren et al., 2003; Hileman et al., 2005; Figure 3). Ekengren et al. (2003) poured tomato seedlings immersed in the *Agrobacterium* infection by turning it upside down into a vacuum device and successfully silenced the *NPR1* and *TGA* genes. Then, Hileman et al. (2005) submerged the seedlings of different development stages into mixtures of *Agrobacterium* cultures and vacuum infiltrated poppy for 1 min, obtaining *PapsPDS* gene-silenced plants.

By vacuum infiltrating germinating wheat and corn seeds under a new *Agrobacterium* suspension containing a recombinant TRV vector and then co-cultivating with *Agrobacterium* cultures for a period of time, Zhang et al. (2017) successfully produced whole plant *PDS* gene-silenced wheat and corn. That was the first time that vacuum infiltration was implemented in a monocot at the seed imbibition stage. The next year, the same team developed a new agroinfiltration method (seed sock agroinoculation VIGS, SSA-VIGS) by soaking exposed cotton seeds in *Agrobacterium* culture during seed imbibition; they then performed functional analysis of *GhBI-1* in response to salt stress. This was the first application of seed imbibition in dicotyledons (cotton) (Zhang et al., 2018). Seed imbibition avoids the extra use of expensive equipment, and the combination of vacuum infiltration and seed imbibition could be a timesaving method for VIGS in the future and might be applied for large-scale genomic studies in more plants.

### Secondary Infiltration

Scientists have also developed a more complex inoculation procedure that is applicable for high-throughput applications of VIGS (Lu et al., 2003). By involving an intermediate step, researchers obtained a high-titer viral inoculum prepared from *Agrobacterium* infiltrated *N. benthamiana* leaves, and then mechanically inoculated it into plants for gene silencing. Sap inoculation (secondary inoculation) is usually conducted by gentle rubbing inoculated *N. benthamiana* leaf sap resuspended in 0.5 M phosphate buffer into carborundum-dusted Arabidopsis leaves. The delivery of leaf sap into Arabidopsis is usually implemented by leaf injection or sap spraying (airbrush infiltration) (Lu et al., 2003). According to the current researches, sap inoculation can also be used by seed imbibition for barley stripe mosaic virus (BSMV)-mediated VIGS and VOX (virus induced gene overexpression) in *N. benthamiana* and monocots (Yuan et al., 2011; Cheuk and Houde, 2017). These studies have improved researchers’ understanding of secondary inoculation in virus-related gene-functional studies and broadened the application range of genes by the sap inoculation method.

## Important Factors of TRV-VIGS

As an experiment of TRV-VIGS carried out from viral vector selection, recombinant viral vector construction, *Agrobacterium* transformation (*in vivo* transcription of virus genome), the delivery of inoculum into plants, and gene silencing in plants, each step may strongly affect the silencing efficiency of the target gene. Therefore, this part discusses the influential factors of TRV-VIGS efficacy to provide reasonable suggestions for researchers.

### Viral Vector Selection

Different viral vectors are suitable for VIGS research in diverse plants with different silencing efficiencies (for details, see Supplementary Table 1). TRV vectors have been widely used for VIGS across various species, both dicot and monocot, due to its advantages of mild viral symptoms and invasion into meristems (Gao et al., 2011; Senthil-Kumar and Mysore, 2011b; Yuan et al., 2011; Liu et al., 2015; Zhang et al., 2017). In *Methods in Molecular Biology*, edited by Courdavault and Besseau (2020), they report that most of the genes in plants, such as chili pepper, diploid potato, flax, and opium poppy, can be silenced with TRV-VIGS protocols, thus providing a better understanding of TRV-VIGS and more choices for gene function analysis in diverse plants. Therefore, TRV might be a really efficient gene silencing viral vector for VIGS, and more studies should be done on its mechanism, methodology, and application.

### Insert Sequence

The optimization of cDNA libraries of the target gene is also very important to the silencing efficiency of TRV-VIGS. Experiments showed that the upper limit of the inserted sequence of viral vectors is about 1500 bp, and the lower limit was determined as 23 nucleotides (nt) identity (Thomas et al., 2001). Introns do not function in guiding VIGS in plants; thus, only exon sequences can be used for VIGS to successfully elicit gene silencing of plant endogenous genes (Ruiz et al., 1998). Liu and Page (2008) gave the following guidelines for constructs of TRV vectors: insert lengths should be in the range of 200–1500 bp and should be positioned in the middle of the cDNA without the homopolymeric regions.

However, the similarity of genes within the gene family also makes simultaneous silencing of multiple genes possible by targeting the conserved gene sequence (Zhou and Zeng, 2017). Multiple genes that are unrelated by nucleotide sequence can be silenced at the same time using VIGS by co-inoculating the respective VIGS constructs or inoculating the recombinant vector with tandem gene inserts (Burch-Smith et al., 2006). Therefore, to perform silencing of a single and specific gene, the insert must be selected according to the specific domain of the gene, or VIGS can be conducted by using the conserved sequence within a gene family to study the function of the whole gene family or in tandem gene inserts.

### *Agrobacterium* Strain

The optimal *A. tumefaciens* strain used for VIGS varies with different plants and also affects the gene silencing efficiency. Studies have shown that *Agrobacterium* strain GV2260 works best in *N. benthamiana*, while strain GV3101 could also be used (Liu et al., 2002a). GV3101 works best for the silencing of TRV-VIGS in tomato, whereas LBA4404 and GV2260 could also be used, but the silencing efficiency is very low (Liu et al., 2002a; Dinesh-Kumar et al., 2003). Both *Agrobacterium* strain GV3101 and AGL-1 with TRV-*MePDS*-infiltrated distal leaves showed an albino phenotype at 20 days post-inoculation (dpi) (Zeng et al., 2019). A suitable temperature for the growth of *Agrobacterium* should be at or below 28°C for no longer than 2 days.

### Inoculum Concentration

As the agroinoculation methods and characteristics of diverse infected plants are different, the concentration of the infection solution strongly affects the gene silencing efficiency of VIGS experiments. The solution used for the resuspension of concentrated *Agrobacterium* should be fresh for each use, and lower concentrations of the inoculum also work, but those higher than 1.0 OD_600_ may cause necrosis on the infiltrated *N. benthamiana* leaves (Dinesh-Kumar et al., 2003). Liu et al. (2002b) found the concentration (OD_600_ = 1.0) of *Agrobacterium* cultures that gave the best *SlPDS* gene silencing results in tomato, while Dinesh-Kumar et al. (2003) found that OD_600_ = 1.5 worked better for tomato. Therefore, for *N. benthamiana*, the OD_600_ should be adjusted to 1.0 and no more than 1.5 for tomato. Burch-Smith et al. (2006) proposed that the most suitable concentration of *Agrobacterium* infection solution is OD_600_ = 1.5 for TRV-VIGS experiment in Arabidopsis with an efficiency of almost 100%. Additionally, the naked cotton seeds soaked in *Agrobacterium* cultures with an OD_600_ of 1.5 for 90 min exhibited an optimal silencing efficiency of SSA-VIGS (Zhang et al., 2018). For vacuum infiltration, Zhang et al. (2017) found that the optimal condition for VIGS was vacuum treatment for 30–60 s with an *Agrobacterium* culture of OD_600_ = 0.3 and co-cultivation with the same concentration of *Agrobacterium* culture for 15 h. These experiments showed that different species require different concentrations of *Agrobacterium* inoculum; thus, the *Agrobacterium* inoculum concentration of each specific TRV-VIGS experiment should be optimized.

### Environmental Factors

Studies have shown that environmental factors directly affect plant growth status and also virus accumulation and spread in plants, which are closely related to gene silencing efficiency and gene silencing duration (inheritance) of TRV-VIGS (Senthil-Kumar and Mysore, 2011b). Therefore, it is necessary to strictly control plant care when conducting VIGS experiments (Burch-Smith et al., 2004).

#### Age or Development Stages of Infected Plants

One study showed that Arabidopsis seedlings inoculated at the two- to three-leaf stage and grown under 16-h light displayed the photo-bleaching phenotype indicative of *AtPDS* silencing in almost 100% of the cases examined, while older seedlings inoculated at the same conditions exhibited reduced *AtPDS* transcript levels (95%) in the silenced plants (Burch-Smith et al., 2006). Studies also indicated that the four-leaf stage in *N. benthamiana* and two-leaf stage in tomato are the optimal ages for syringe infiltration of VIGS experiments. Another TRV-VIGS study in *Streptocarpus rexii* (*Gesneriaceae*) showed that the efficacy of TRV-VIGS showed a correlation with the age of *S. rexii* seedlings (Burch-Smith et al., 2006). The younger the plants were when infected with TRV, the more efficient the silencing of the *SrPDS* gene (Nishii et al., 2020).

#### Ambient Temperature

Previous reports showed that temperature is a key player in influencing the gene silencing phenotype development in plants with VIGS (Szittya et al., 2003; Fu et al., 2006; Tuttle et al., 2008). Generally, the gene silencing efficiency of TRV-VIGS was significantly reduced under high temperatures (temperatures higher than 27°C dramatically reduced virus titer levels in plants), while virus concentration and gene silencing efficiency were largely increased at low temperature (16–21°C for tomato) (Szittya et al., 2003; Caplan and Dinesh-Kumar, 2006). The most important reason for the success of the first long gene silencing report (duration of more than 2 years, and the acquisition of gene silencing T1 and T2 generation offspring) of TRV-VIGS is good plant care under low temperatures (18–20°C) (Senthil-Kumar and Mysore, 2011b). Therefore, efficient TRV-VIGS experiments should be conducted at relatively lower ambient temperatures (below 21°C for tomato) and then shifted to the optimum temperature for plant growth (24°C for tomato) after 1 or 2 days (Caplan and Dinesh-Kumar, 2006).

#### Humidity

An appropriate humidity is also beneficial for the silencing efficiency of TRV-VIGS. Fu et al. (2006) found that when cultured at a lower humidity (30–40%), more than 90% of tomatoes exhibited a *SlPDS* silenced phenotype. At the same time, they also found that TRV-RNA (RNA1 and RNA2) was more efficiently introduced into flowers and fruits at a lower humidity.

Therefore, good plant care (appropriate ambient temperature and humidity, adequate water and fertilizer, and free of pests and diseases) to maintain efficient silencing and healthy growth of gene-silenced plants is important for VIGS experiments and should be optimized in every single VIGS system.

### Positive Controls

Tobacco rattle virus-VIGS can effectively downregulate the expression of *PDS*, *H subunit of magnesium chelatase* (*ChlH*), anthocyanidin synthase (*ANS*), and other genes in *Solanaceae* plants with distinct gene silencing phenotypes (Senthil-Kumar et al., 2008); thus, they are often utilized as positive controls to optimize and determine the efficacy of a VIGS system. For instance, Liu et al. (2002a) calculated the silencing efficiency of TRV-VIGS in tomato plants with *SlPDS* as a positive control (silencing plant with an albino phenotype) by leaf injection (50%) and *Agrobacterium* spray (90%). The *transparent testa 2* (*TT2*) gene might be a positive control for genes that are expressed in seeds (seed coat). Seed bolls (containing seeds) used for TRV-mediated VIGS of *TT2* gene showed a result of testa depigmentation with a silencing efficiency around 35% in flax seeds (*Linum usitatissimum* L.) (Hano et al., 2020). Only with an optimized positive control for each specific silenced region (organ) can a VIGS system provide the best silencing efficiency for application studies in that species. Thus more positive controls should be found and determined for some specific regions in VIGS system, such as root or other organs.

### Inoculation Method

To realize the gene functions across different plant development stages, researchers have developed many VIGS inoculation methods (Figure 3). Leaf injection is the most commonly used inoculation method to silence genes related to leaf development, floral development, fruit development, metabolic processes, and plant-pathogen interactions (R genes) in different plants (Ratcliff et al., 2001; Liu et al., 2002a,b, 2004; Chen et al., 2004; Ryu et al., 2004). When studying genes related to seedling development, floral development, or other fields of monocot plants, *Agrobacterium* spray, agrodench, and sap inoculation can be used for VIGS of target genes (Scofield et al., 2005; Cloutier et al., 2007; Zhang et al., 2017). For tomato genes, using spray infiltration, the silencing success rate was about 90% (Dinesh-Kumar et al., 2003). For gene-functional analysis of root development, seed dormancy, and seed germination, the preferred methods are agrodench, vacuum infiltration, and seed imbibition (Zhang et al., 2017; unpublished data).

Tobacco rattle virus-VIGS studies showed that agrodench coupled with leaf inoculation provoked an effective gene silencing phenotype in tomato and tobacco plants that lasted for more than 2 years, and gene silencing was maintained in T1 and T2 generation offspring (Senthil-Kumar and Mysore, 2011b). Combined sap inoculation and seed imbibition, Cheuk and Houde (2017) also obtained better and more even gene expression levels of the BSMV homogenate than other VOX experiments conducted by leaf injection or leaf friction of leaf sap. Therefore, an integrated application of different inoculation methods might trigger better gene silencing results using VIGS.

**FIGURE 4:**
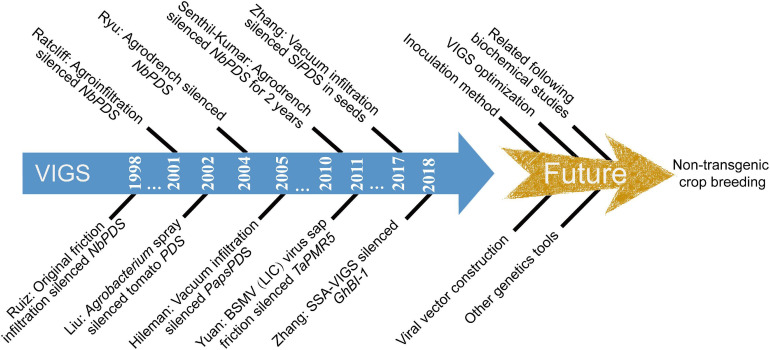
Important historical milestones of VIGS system. TRV: Tobacco rattle virus; BSMV: Barley stripe mosaic virus; LIC: Ligation-independent cloning; SSA-VIGS: Seed sock agroinoculation VIGS; NbPDS: Tobacco phytoene desaturase; TaPMR5: Wheat powdery mildew resistance 5 gene; SlPDS: Tomato phytoene desaturase; GhBI-1: Cotton Bax inhibitor-1.

These studies provide an important reference for the improvement and application of TRV-VIGS. By the comprehensive comparison and combination of different agroinoculation methods (multiple methods conducted together) under appropriate plant care, researchers can create a more suitable VIGS method for functional genomic analysis (Figure 4).

## Applications of TRV-VIGS Technology

With the improvement of VIGS tools, TRV-VIGS has been utilized for the functional genomic analysis of more and more plant species, including most dicotyledonous species, some monocotyledonous plants, and even some trees. The gene function of many genes in different plant tissues and organs, from seeds, roots, stems, leaves, flowers, and fruits, have been revealed by TRV-VIGS in the past 20 years that relate to plant growth, development and reproduction, metabolic pathways, and response to biotic and abiotic stress, among others (Burch-Smith et al., 2004; Purkayastha and Dasgupta, 2009; Becker and Lange, 2010). A complete review of the literature on all aspects of VIGS application is out of the scope of this work; here, this article attempts to provide an update on the most recent applications of VIGS in plants.

### Dicotyledon

#### Solanaceae

Since TRV-VIGS was first derived from tobacco, it is widely used for functional gene studies in other solanaceous plants (Purkayastha and Dasgupta, 2009; Senthil-Kumar and Mysore, 2011a, 2014). VIGS has been used to study the functional roles played by several kinases, such as (*NPK1*, *WIPK*, and *SIPK*, in defense against TMV-mediated by *N*-gene resistance in *N. benthamiana* (Jin et al., 2002, 2003; Sharma et al., 2003; Liu et al., 2004). The TRV-VIGS study of *RanGAP2* showed that the interaction with Rx (an NB-LRR protein from potato) is required for conferring PVX resistance in *N. benthamiana* (Tameling and Baulcombe, 2007). VIGS silencing of two genes, an extracellular peroxidase and a cytochrome P450, revealed their defense-related roles in pepper (Kim et al., 2006; Choi et al., 2007). Another VIGS study demonstrated the role of a gene encoding arabinogalactan protein, attAGP in tomato during the attack by plant parasite *Cuscuta reflexa* (Albert et al., 2006). The TRV-mediated VIGS system has been applied for gene-functional analysis in eggplant (Liu et al., 2012). VIGS silencing of the *VTE1* gene in tomato leaves with an *Agrobacterium* infection solution carrying the TRV vector demonstrated that *VTE1* is involved in vitamin E synthesis in tomatoes (Lu et al., 2011). Organ aging issues in petunia flowers and functional analysis of peony genes have also been demonstrated with TRV-VIGS technology (Chen et al., 2005; Xie et al., 2019).

Virus-induced gene silencing has also been used for the functional analysis of root genes. Silencing of genes in roots is more effective by the agrodrench method than by syringe infiltration (Ryu et al., 2004). The functional roles of *IRT1* (iron-regulated metal transporter), *TTG1* (*transparent testa glabra*), *RHL1* (*root hairless1*), β-tubulin, *RML1* (*root meristemless1*), and nematode resistance (*Mi*) genes, which are involved in root development, have been demonstrated by this vector (Jablonska et al., 2007).

Recently, VIGS has unraveled the functional redundancy among group III members in their requirements for plant development and plant immunity-associated ROS production (Zhou and Zeng, 2017). Functional analysis of antiviral resistance signaling pathways has also been demonstrated by TRV-VIGS in *N. benthamiana* (Hashimoto et al., 2019). The TRV-mediated VIGS system was established in 4-week-old *in vitro* diploid potato plants by syringe infiltration (Zhao et al., 2020). Additionally, TRV- and PVX-based microRNA silencing (VbMS) approaches to silence endogenous miRNAs in *N. benthamiana* and tomato plants by *Agrobacterium* infiltration have also been developed, thus facilitating functional studies of miRNAs in plants (Zhao et al., 2020).

#### Cruciferae

With the development of VIGS technology, many gene-functional studies have been done in the model plant Arabidopsis of the Cruciferae family (Senthil-Kumar and Mysore, 2011a). Cai et al. (2006) reported some factors that potentially influence TRV-mediated VIGS of *PDS* and actin gene expression in Arabidopsis. In addition, the optimized procedure (by *PDS* gene silencing) for the TRV-based VIGS is a potentially powerful tool for deciphering the signal transduction pathways of disease resistance in Arabidopsis (Burch-Smith et al., 2006; Cai et al., 2006). VIGS experiments carrying the *R* gene *rps4* were conducted in Arabidopsis and demonstrated that the cell death, upon infection with *Pseudomonas syringae*, is dependent on three plant signaling components, EDS1, SGT1, and HSP90 (Zhang et al., 2004).

#### Malvaceae

The most important commercial crop of the Malvaceae family, cotton has also adopted the TRV-VIGS system for functional genomics studies. VIGS silencing of *GhNDR1* and *GhMKK2* by cotyledon injection compromised cotton resistance to *Verticillium wilt* (Gao et al., 2011). The successfully functional characterization of *KATANIN* and *WRINKLED1* in cotton provided evidence that the TRV-VIGS system can be used for rapid functional analysis of genes involved in cotton fiber development (Qu et al., 2012). SSA-VIGS is a powerful tool for elucidating the functions of genes (*GhBI-1* gene, in response to salt stress) in cotton, especially for genes expressed in young seedlings and roots (Zhang et al., 2018). Knockdown of *Gh_A05G1554* (*GhDHN_03*) and *Gh_D05G1729* (*GhDHN_04*) dehydrin genes has revealed their potential role in enhancing osmotic and salt tolerance in cotton (Kirungu et al., 2020). Therefore, combining cotyledon injection together with the SSA-VIGS system (Zhang et al., 2018), almost all cotton genes can be characterized via VIGS assays in the future.

#### Leguminosae

Tobacco rattle virus-VIGS has also been used to study the functional roles of disease resistance genes in soybean. Through the use of VIGS assays in soybean, Liu et al. (2015) studied the relationship between TRV-VIGS technology and soybean mosaic virus (SMV) and demonstrated that resistance to SMV is not affected by early TRV inoculation in soybean.

#### Fruit Trees

The TRV-VIGS system has also been widely used for functional gene studies in fruit trees, including peach, cherry, strawberry, and litchi (Jia et al., 2010; Li et al., 2015, 2016; Xie et al., 2020). VIGS of the *PpChlH* gene proved that this gene plays a role in the synthesis of chlorophyll in peach leaves and fruits (Jia et al., 2010). VIGS silencing of the *PacCYP707A1* (8′-hydroxylase) gene resulted in the silenced-cherries responding to dehydration during fruit development when adjusting ABA in cherries (Li et al., 2015). Similarly, *UFGT1* (UDP-glucose: flavonoid 3-O-glycosyltransferase) plays a key role in the formation of litchi red peel, as documented by VIGS experiments (Li et al., 2016).

#### Ornamental Plants

A study demonstrated that the TRV-based VIGS technique could be adapted for high-throughput functional characterization of genes in the perennial tree peony (Xie et al., 2019). Nishii et al. (2020) utilized the broad host range TRV vector to target the *SrPDS* gene of *S. rexii* (*Gesneriaceae*) by agroinfiltration and sap inoculation and successfully obtained *SrPDS* silenced plants.

#### Other Commercial and Medicinal Plants

Moreover, several non-model plants, such as important commercial and medicinal plants, including cassava, flax, Papaver, *Antirrhinum*, mint, sweet basil, Ashwagandha, and olive tree, were also successfully explored with the TRV-VIGS system. Compared with the obvious cassava mosaic disease symptoms infiltrated by African cassava mosaic virus (ACMV)-based VIGS systems in previous studies, the TRV-mediated VIGS system in cassava plants showed mild disease symptoms, thus suggesting that the application of the TRV-VIGS system could promote functional genomics in cassava (*Manihot esculenta* Crantz) with a significant advantage (Zeng et al., 2019). A detailed protocol has been presented to perform TRV-VIGS assay by agroinoculation in cassava (Zaidi et al., 2020). Two detailed *Agrobacterium*-mediated infection protocols have also been described in flax (*L. usitatissimum* L.), based on whole plant vacuum and leaf syringe infiltration methods. The systemic impact on the gene transcript levels in the stem demonstrated that the VIGS system can be applied for the functional study of cell wall genes in flax (Chantreau and Neutelings, 2020).

Chen et al. (2020) investigated genes involved in alkaloid biosynthesis metabolism in opium poppy using syringe infiltration. Cruz et al. (2020) described the main steps of this biolistic (delivery of the transforming plasmids through a particle bombardment)-mediated TRV-VIGS in *Catharanthus roseus* and *Rauvolfia tetraphylla* of *Apocynaceae*. A recent report showed that TRV-VIGS can be used as a rapid gene function test for the complementation of a loss-of-function mutation in *Antirrhinum majus* L. (Tan et al., 2020). VIGS technology has also been established and adapted to target genes involved in the production of nepetalactone in *Nepeta cataria* (catnip) and *Nepeta mussinii* (catmint) of the Lamiaceae family (mint) (Palmer and O’Connor, 2020). A robust protocol for VIGS of sweet basil (*Ocimum basilicum*) *ObChlH* has recently been developed by vacuum infiltration with pTRV constructs (Misra et al., 2020). As a highly recalcitrant plant for genetic transformation, Ashwagandha (*Withania somnifera*), an important Indian medicinal plant, has an established procedure to carry out VIGS for gene function studies (Bomzan et al., 2020). TRV-mediated VIGS through agroinoculation of olive plantlets has been successfully performed for functional genomic analyses in the olive tree (Oleaceae) (Koudounas et al., 2020).

### Monocotyledon

Not only can the TRV-VIGS system be used in most dicotyledonous species, it has also been adopted for gene-functional analysis of some monocotyledonous plants that are susceptible to TRV, such as wheat, corn, barley, rice, and *Brachypodium sylvaticum* in Gramineae and ornamental plants, such as orchids, a flowering plant of Orchidaceae.

#### Gramineae

Virus-induced gene silencing has been successfully applied for functional characterization of genes involved in leaf rust resistance of wheat (Scofield et al., 2005; Cloutier et al., 2007). Similarly, vacuum and co-cultivation agroinfiltration of (germinated) seeds resulted in TRV-mediated whole-plant *TaPDS* and *ZmPDS* gene silencing in wheat and maize (Zhang et al., 2017). These results proved that this system is suitable for functional analysis of genes involved in seed germination and early plant development stages (Zhang et al., 2017). Additionally, with the VIGS system, Li et al. (2019) demonstrated that the MAC3 (MOS4-associated complex) protein is a key regulatory factor necessary for corn endogenous immunity and disease resistance.

#### Orchidaceae

Virus-induced gene silencing has also been adapted for functional validation of genes involved in floral growth and development of orchids (Hsieh et al., 2013). Chen et al. (2020) successfully silenced the *PoFYF1/2* gene in Phalaenopsis orchids and revealed that *PoFYF1/2* plays a role in suppressing senescence/abscission during early flower development.

## Limitations

Virus-induced gene silencing is a promising genetic tool for functional genomic studies in plants. However, like any other technique, there are still some limitations of TRV-VIGS system. First, the timing of VIGS appearance, as well as gene silencing duration, is usually species-specific. The first long silencing duration report of the TRV-VIGS system in tobacco and tomato (Senthil-Kumar and Mysore, 2011b), together with other long silencing duration reports of BSMV-VIGS in wheat (Bruun-Rasmussen et al., 2007) and Apple latent spherical virus (ALSV)-VIGS in soybean (Igarashi et al., 2009; Yamagishi and Yoshikawa, 2009), will surely provide significant promise for the application of VIGS system in studies related to perennial crop breeding (Senthil-Kumar and Mysore, 2011a). So researchers should continue to prolong the gene silencing period of VIGS in the future.

Another challenge of this technique is its varying penetrance of the phenotype in vegetative and reproductive tissue that requires a larger number of plants to be screened for phenotypes. So far, studies have shown that silencing efficacy is often found regionally, dividing the whole plant or restricted to plant gene silenced regions without a few consecutive nodes (Wege et al., 2007; Becker and Lange, 2010). Thus, proper positive control selection and a combination of different inoculation methods are necessary for an efficient VIGS system in diverse tissues at different developmental stages of crops. However, most known positive controls are always visual phenotype of photo-bleaching on leaves, without any marker genes in other silenced regions. Hence, more positive control genes in different tissues should be screened in future VIGS system.

Additionally, the stability of the environmental control and operation techniques is also worth noting in future VIGS studies, because the environment can cause changes in the silencing effect. For example, compared with the BSMV-VIGS assay in wheat and TRV-VIGS in *N. benthamiana*, reports showed that the Chinese wheat mosaic virus (CWMV) vector is more effective in silencing endogenous genes and miRNAs at 17°C, thereby providing a powerful tool for gene function analysis in both *N. benthamiana* and wheat to fulfill the functions of the VIGS system at low temperatures (Yang et al., 2018). Since VIGS assay involves viruses that can be easily transmitted to other plants in a field environment, VIGS vector carrying inocula, seeds and other plant materials should be disposed under suitable biosafety regulation (Senthil-Kumar and Mysore, 2011a). What’s more, although TRV has a wide host range, it still could not infect all plants to some extent. Therefore, it is one of the important content for future researches to develop new VIGS vectors and also to modify the existing vectors to increase its host range and silencing efficiency (Senthil-Kumar et al., 2008). Note that many of these limitations are inherent to all VIGS vectors and are not specific to TRV.

## Conclusion and Future Prospects

In the past two decades, many VIGS vectors have been developed (Supplementary Table 1), and TRV was preferentially used for VIGS assays in most dicots and some monocots, due to the high susceptibility of a wide range of hosts with mild viral symptoms after infection (Chen et al., 2005; Zhang et al., 2017; Xie et al., 2019). The increase in high-quality genome or transcriptome (EST) data has provided an excellent foundation and sufficient homologous sequence information for VIGS assays to target a specific gene, gene family, gene class, or miRNA mimic (Senthil-Kumar and Mysore, 2011a; Figure 1). As viral vector construction of TRV is an important factor of an efficient VIGS system, many modifications have been done based on the original TRV vector to obtain better silencing efficiency in different species (Figure 2). Multiple inoculation methods for introducing the viral constructs into plants have also been developed to extend the application range of genes that can be studied by this system (Figure 3). With the development of VIGS studies, scientists have found that the silencing efficiency of the VIGS system is strongly affected by the selection of *Agrobacterium* strain, inoculum concentration, environmental factors (plant care), and positive control; thus, many established VIGS protocols have been optimized. As VIGS protocols can be transposed to many other plants following minor adaptation, recently established TRV-VIGS protocols in many species will be a great help in deepening researchers’ understanding of gene functions in a variety of plant species (Courdavault and Besseau, 2020).

In total, all improvements of VIGS technology will speed up the application of this tool for identifying candidate genes involved in various aspects of plant biology (Figure 4), including plant-environment interactions, plant growth and development, metabolic processes, and other cellular processes *in planta* (Senthil-Kumar et al., 2008). A deeper understanding of the VIGS mechanism has provided researchers with the option of combining VIGS with other functional genomic approaches (conventional or molecular breeding and next-generation technology) for crop breeding studies (Cheng et al., 2010; Senthil-Kumar and Mysore, 2011a). VIGS can also be performed in a stable plant that overexpresses, downregulates (RNAi), or knocks out (mutant) an unrelated gene compared with the gene targeted by VIGS to characterize the function of a genetic pathway (Baena-Gonzalez et al., 2007).

In conclusion, a good VIGS system should be established with proper viral vector construction, inoculation method, optimized inoculum type and concentration, proper positive controls with good plant care and plant vigor, thus eliciting high gene silencing efficiency with a uniform phenotype and simple operation procedure with lower equipment requirements and largely extending the scope of genes that can be studied (Liu et al., 2002a; Benedito et al., 2004; Ryu et al., 2004; Yuan et al., 2011; Zhang et al., 2017; Cheuk and Houde, 2018). Finally, a proper VIGS assay together with other genetic tools and related biochemical studies (Lu et al., 2003) will surely provide a promising future for non-transgenic crop breeding with high production in diverse plants.

## Author Contributions

GS, ZX, and MH researched data for the article, substantially contributed to discussion of the content, and wrote the manuscript. ZX, FW, BT, and GC substantially contributed to discussion of the content and reviewed the manuscript before submission. All authors have read and approved the manuscript to be published.

## Conflict of Interest

The authors declare that the research was conducted in the absence of any commercial or financial relationships that could be construed as a potential conflict of interest.
